# Suicide Prevention: A Handbook for Community Gate Keepers

**Published:** 2009

**Authors:** R. C. Jiloha

**Affiliations:** Department of Psychiatry, GB Pant Hospital, Maulana Azad Medical College and Faculty of Medical Sciences, University of Delhi, Delhi, India. E-mail: rc.jiloha88@gmail.com

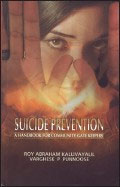



Approximately 1 million people commit suicide every year worldwide, of which more than 1 lakh belong to India, and approximately 10 times of this figure people attempt suicide per year. Suicide is one of the most baffling subjects, that human beings have an ancient tradition of self-murder. Suicide is a major public health problem throughout the world, and one for which major efforts are being made to help reduce these numbers by those engaged in this field. However, suicide is the result of complex interactions between a range of factors, including psychological, cultural, biological and social. Any approach to treating the problem of suicide has to consider all these factors.

The book, Suicide Prevention: A Handbook for Community Gate-Keepers, is one such attempt in this direction. It is a comprehensive book on suicide prevention. This volume presents an overview of the current scientific knowledge about suicide and suicide prevention. Contributed by multiple authors, the book comprises 28 chapters, covered in 167 pages. Multidisciplinary and comprehensive in scope, the book provides a solid foundation in theory, research and clinical applications. The issues related to suicide assessment, methodological profile and clinical case management are highlighted and various preventive modalities are discussed in the light of the latest research findings. The topics covered range from cause, risk factors and their assessment to religious, sociocultural and ethical paradigms related to suicide. It also talks of vulnerable groups of population such as adolescents, women and the elderly, who are afflicted by suicide. It lays extra emphasis on suicide prevention by devoting four chapters exclusively to the preventive aspect of suicide. It presents an overview of research within disciplines covering all factors, including psychological, cultural, biological and sociological. The book provides up-to-date information on different kinds of risk and protective factors, epidemiology, theories on suicidal behaviors and public health and healthcare preventative approaches. In the last chapter, the book highlights the techniques to report a suicide case. Written by well-known authorities on suicide, the book also includes contributions of other leading scholars who report on their respective area of expertise. Throughout, a user-friendly book, its annotated references enhance the volume's utility to clinicians, students and the public in general.

